# Glucose Induces ECF Sigma Factor Genes, *sigX* and *sigM*, Independent of Cognate Anti-sigma Factors through Acetylation of CshA in *Bacillus subtilis*

**DOI:** 10.3389/fmicb.2016.01918

**Published:** 2016-11-29

**Authors:** Mitsuo Ogura, Kei Asai

**Affiliations:** ^1^Institute of Oceanic Research and Development, Tokai UniversityShizuoka, Japan; ^2^Department of Bioscience, Saitama UniversitySaitama, Japan

**Keywords:** protein lysine acetylation, nutritional signal, transposon mutagenesis, sigma factor, RNA polymerase

## Abstract

Extracytoplasmic function (ECF) σ factors have roles related to cell envelope and/or cell membrane functions, in addition to other cellular functions. Without cell-surface stresses, ECF σ factors are sequestered by the cognate anti-σ factor, leading to inactivation and the resultant repression of regulons due to the inhibition of transcription of their own genes. *Bacillus subtilis* has seven ECF σ factors including σ^X^ and σ^M^ that transcribe their own structural genes. Here, we report that glucose addition to the medium induced *sigX* and *sigM* transcription independent of their anti-σ factors. This induction was dependent on an intracellular acetyl-CoA pool. Transposon mutagenesis searching for the mutants showing no induction of *sigX* and *sigM* revealed that the *cshA* gene encoding DEAD-box RNA helicase is required for gene induction. Global analysis of the acetylome in *B. subtilis* showed CshA has two acetylated lysine residues. We found that in a *cshA* mutant with acetylation-abolishing K to R exchange mutations, glucose induction of *sigX* and *sigM* was abolished and that glucose addition stimulated acetylation of CshA in the wild type strain. Thus, we present a model wherein glucose addition results in a larger acetyl-CoA pool, probably leading to increased levels of acetylated CshA. CshA is known to associate with RNA polymerase (RNAP), and thus RNAP with acetylated CshA could stimulate the autoregulation of *sigX* and *sigM*. This is a unique model showing a functional link between nutritional signals and the basal transcription machinery.

## Introduction

The association of σ factor with RNAP determines the specific binding of the RNAP holoenzyme to target promoters (Helmann and Chamberlin, 1988). The varieties of σ factors enhance the chance for survival in the environment via fine-tuning of the regulation of gene expression. Bacteria have several distinct σ factors including extracytoplasmic function (ECF) σ factors, which are composed of large paralog gene families (Souza et al., 2014; Helmann, 2016). ECF σ factors commonly respond to cell envelope stresses and transcribe genes related to the stress response (Souza et al., 2014; Helmann, 2016). In *Bacillus subtilis*, seven ECF σ factors, σ^M^, σ^V^, σ^W^, σ^X^, σ^Y^, σ^Z^, and σ^Y laC^ are encoded in the genome (Helmann, 2016). In many cases, these σ factors recognize similar nucleotide sequences. σ^M^, σ^V^, σ^W^, and σ^X^ bind to the consensus -35 and -10 elements, (T)GAAACNT and CGT(C/A)T, respectively (Helmann, 2016). As a result, these σ-factor regulons overlap, yet there are genes transcribed by a single ECF σ factor. σ^X^- and σ^M^-RNAPs transcribe their own genes: *sigX* and *sigM*, respectively (Asai et al., 2003; Helmann, 2016). The σ^X^ regulon contains the genes required for resistance against the antibiotic nisin and the peptidoglycan synthesis inhibitor bacitracin, and the genes encoding enzymes for D-alanylation of teichoic acids, which are required for the resistance against cationic antimicrobial peptides (Huang and Helmann, 1998; Cao and Helmann, 2002, 2004; Asai et al., 2003). In the *sigX* mutant, levels of heat and oxidative stress resistance are low, but the mechanism responsible for this is not yet known (Huang et al., 1997). The σ^M^ regulon includes >60 genes such as core genes for cell wall biogenesis and cell division (*rodA, divIC, mreBCDminCD*, and *murBdivIB*), regulatory genes (*spx* and *abh*), and the bacitracin resistance gene *bcrC* (Cao and Helmann, 2002; Thackray and Moir, 2003; Eiamphungporn and Helmann, 2008; Luo and Helmann, 2009).

In most cases, a gene encoding an ECF σ factor is associated with a gene encoding a corresponding anti-σ factor (Ho and Ellermeier, 2012). The anti-σ factor embedded in the cell membrane traps the cognate σ factor through a protein–protein interaction, leading to repressed expression of the regulon. A detailed mechanism for the release of σ factor from anti-σ factor in *B. subtilis* is well-understood for σ^W^ and includes specific proteolysis of its cognate anti-σ factor (Ho and Ellermeier, 2012). Exposure to antibiotics that interfere with cell wall biosynthesis induces ECF σ factors (e.g., the peptidoglycan synthesis inhibitor vancomycin induces *sigM*) (Thackray and Moir, 2003). In addition, some mutations in the cell envelope biosynthesis pathway induce the *sigX* and/or *sigM* regulons (Hashimoto et al., 2013). For example, a mutation in *yfhO*, encoding a probable flippase for polymers synthesized by the CsbB glucosyltranfserase, enhanced *sigM* regulon expression (Inoue et al., 2013). A mutation in *ugtP*, which encodes a UDP glucosyltransferase, induces expression of the σ^X^ regulon (Matsuoka et al., 2011). Transposon mutagenesis for mutants that upregulate *sigX* promoter activity, that is, screening for inhibiting genes of σ^X^ activity, resulted in identification of seven mutations including a multidrug efflux pump gene (Turner and Helmann, 2000).

In this study, we identified that protein lysine acetylation is involved in *sigX* and *sigM* regulation. Protein lysine acetylation is a well-conserved protein modification in both eukaryotes and prokaryotes (Wang et al., 2010; Thao and Escalante-Semerena, 2011; Bernal et al., 2014). In prokaryotes, including *B. subtilis*, several global analyses of protein lysine acetylation have been reported, although the functional analysis of each gene was poorly performed (Kim et al., 2013; Kosono et al., 2015; Carabetta et al., 2016).

Spx is a global regulatory protein with a role in the disulfide stress response and the other cellular processes (Zuber, 2004; Rochat et al., 2012). Spx is bound to α-subunit of RNAP. We observed glucose induction (GI) of the *spx* gene in sporulation medium (Shiwa et al., 2015). Another group has also reported GI of the *spx* regulon in LB medium supplemented with glucose (Yang et al., 2014). Here, we report that glucose addition to the medium induced *sigX* and *sigM* transcription independent of their anti-σ factors. The GI of *spx* was caused by the GI of *sigX* and *sigM*. The induction would be dependent on the cellular acetyl-CoA pool. Transposon mutagenesis for the mutant showing no induction of *sigX* and *sigM* revealed that *cshA*, which encodes DEAD-box RNA helicase, is required for induction (Lehnik-Habrink et al., 2010). According to the global acetylome analysis in *B. subtilis*, CshA has two lysine residues that can be acetylated (Kosono et al., 2015). We showed that in the *cshA* mutant with acetylation-abolishing K to R exchange mutations, GI of *sigX* and *sigM* was abolished and that glucose addition stimulated acetylation of CshA. Thus, we present a model in which glucose addition results in a larger acetyl-CoA pool, probably leading to an increase in acetylated CshA. CshA is known to be associated with RNAP (Delumeau et al., 2011), and thus RNAP with acetylated CshA could stimulate autoregulation of *sigX* and *sigM*.

## Materials and Methods

### Strains, Media, and β-Galactosidase Analysis

All of the *B. subtilis* strains used in this study are listed in Supplementary Table S1. One-step competence medium (Kunst et al., 1994) [MC], Schaeffer’s sporulation medium (Schaeffer et al., 1965), and Luria-Bertani (LB) medium (Difco, Lennox) were used. Antibiotic concentrations were described previously (Ogura and Tanaka, 1996; Ogura et al., 1997). Synthetic oligonucleotides were commercially prepared by Tsukuba Oligo Service (Ibaraki, Japan) and are listed in Supplementary Table S2.

### Growth Condition

Strains were grown on a LB agar plate (1.5%) containing appropriate antibiotics at 37°C overnight. The cells were scraped from the plate and suspended in the sporulation medium. The suspension was inoculated into 50 ml sporulation medium (with or without glucose) without antibiotics in a 200-ml flask. Klett value was adjusted around 10 units. The flask was gently shaken (110 reciprocation/min) at 37°C. Cell growth was monitored using Klett colorimeter (Klett Mfg., Co., Inc., New York, NY, USA).

### Plasmid Construction

The plasmids used in this study are listed in Supplementary Table S1. pIS284-sigM-del1 and pDG1663-sigX-del2 were constructed by cloning of the double-stranded oligonucleotides PsigM-F/PsigM-R and PsigX-F/PsigX-R into pIS284 and pDG1663 which were treated with EcoRI/BamHI, respectively (Guérout-Fleury et al., 1996; Tsukahara and Ogura, 2008). To construct pDG1663-sigX-Wt and pDL2-sigX-del1, PCR products amplified by using the oligonucleotide pair SigX-F/SigX-R and SigX-F/SigX-R2, respectively, were digested with EcoRI/BamHI and cloned into pDG1663 and pDL2 treated with the same enzymes (Yuan and Wong, 1995). To construct pDG1729-PcshA, PCR products amplified by using the oligonucleotide pair, pDG1729-PcshA-E/pDG1729-PcshA-H, were digested with EcoRI/HindIII and cloned into pDG1729 treated with the same enzymes (Guérout-Fleury et al., 1996). To construct pMUTIN3DZ, PCR products amplified by using the oligonucleotide pair, ypuN-F/ypuN-R, were digested with HindIII/BamHI and cloned into pMUTIN3DZ treated with the same enzymes (Yoshimura et al., 2004).

### Construction of Strains

The *bkdB*::Pxyl-*cshA* unit was constructed using PCR (Supplementary Figure S1). The *bkdB*-up linked to Km^r^ and *xylR*-Pxyl units were amplified from the total DNA of the strain containing *bkdB*::*sinR* and the pX plasmid, respectively (Hori et al., 2002; Ogura, 2016). The other fragments were amplified from the wild type derived total DNA. PrimeSTAR MAX DNA polymerase was used (TaKaRa). Four DNA fragments were assembled by using Gibson Assembly Master Mix (New England Biolabs). The sequences of the *cshA* ORF in the resultant strains were confirmed by sequencing using the primers pX-cshA-seq-F and pX-cshA-seq-R. The *cshA*::Tc^r^ unit was constructed using PCR. Briefly the upstream and downstream regions and Tc^r^ from pBEST304 (Itaya, 1992) were amplified using the indicated primers (Supplementary Table S2) and then combined by PCR.

### Sublancin Production Assay

Spot-on-lawn assays were performed as previously reported (Luo and Helmann, 2009). To prepare the lawn, overnight culture of the Sublancin-sensitive Y13 cells in liquid LB medium was inoculated into 4 ml of melted 0.7% LB agar (2%), and poured onto the solid 1.5% LB agar plate. Both media were supplemented with or without 1% glucose. Plates were dried for 30 min in an incubator (37°C), and 3 μl of each overnight culture in liquid LB medium was spotted on plates and incubated at 37°C.

### Transposon Mutagenesis

The transposon delivery vector pMarA (Le Breton et al., 2006) was introduced into the strain OAM709. The resultant strain was grown in liquid LB medium containing kanamycin at 30°C overnight. The cells were diluted and plated onto sporulation medium with 1.5% agar plates containing 2% glucose, X-gal (100 μg/ml), kanamycin, and erythromycin. The plates were incubated at 42°C and the pale colonies were chosen. Otherwise, the diluted cells were plated onto LB medium with 1.5% agar plates containing kanamycin and erythromycin, and incubated at 42°C. Colonies were then transferred onto sporulation medium with 1.5% agar plates containing 2% glucose, X-gal (100 μg/ml), kanamycin, and erythromycin. The plates were incubated at 37°C to choose pale colonies. The insertion mutations were backcrossed into the parental strain and the resultant strains were used for the Lac-assay. Total DNA was taken from the candidate strain, SauIIIA1-digested, self-ligated, and subjected to inverse PCR using oligonucleotides 695 and 696 as described previously (Chan et al., 2014, Supplementary Table S2). The PCR products were sequenced using the oligonucleotide 696.

### Purification of CshA-His

CshA-His was purified using a Ni-affinity column from *B. subtilis* OAM730 (*cshA bkdB*::Pxyl-*cshA*-His) cells as described previously (Ogura et al., 2014).

### Western Blot Analysis

Western blot analysis was performed by a method described previously (Hata et al., 2001). Monoclonal anti-acetylated lysine rabbit antibody was purchased from Cell Signaling Technology (Danvers, MA, USA). This antibody was diluted (1/1000) in 1x TBS with 5% BSA and 0.1% Tween20, and the solution was incubated with a protein-blotted nitrocellulose filter overnight at 4°C. To enhance signal, Can Get Signal solution 2 (ToYoBo) was used for secondary antibody.

## Results

### Abolishment of GI of *spx* in the *sigX* and *sigM* Mutants

**FIGURE 1:**
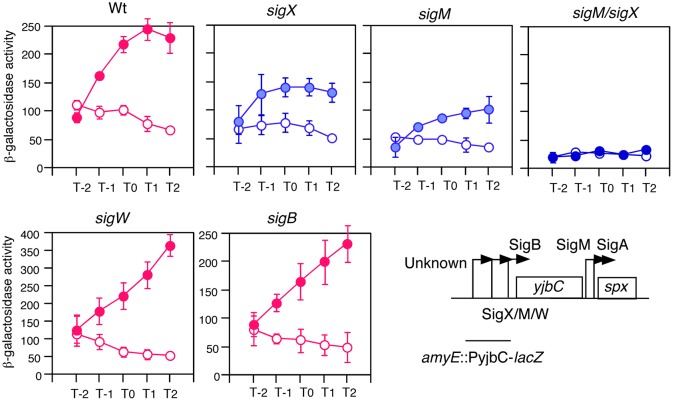
**GI of PyjbC promoter and effects of σ gene disruption.** Cells were grown in sporulation medium with (closed symbols) or without (open symbols) 2% glucose. Data sets showing GI and not showing GI are shown in red and blue, respectively. Averages from at least three independent experiments and standard deviations (error bar) are shown. Cells were sampled hourly. β-galactosidase activities are shown in Miller units. The X-axis represents the growth time in hours relative to the end of vegetative growth (T0, see Supplementary Figure S2). The chromosomal structure of P*yjbC-lacZ* in OAM702 is shown. Each introduced gene disruption into OAM702 is indicated above the panel. Boxes and bent arrows show open-reading frames and promoters, respectively. Text along with the bent arrow show the σ factors responsible for the promoter activity.

We observed GI of the *spx* gene in sporulation medium (Shiwa et al., 2015). The transcription of the *yjbC-spx* operon is complex. Five promoters and five σ factors have been reported to play roles in transcription of the operon (Antelmann et al., 2000; Thackray and Moir, 2003; Cao and Helmann, 2004; Jervis et al., 2007, **Figure 1**). To clarify which σ factors other than σ^A^ are responsible for GI, we introduced several mutations in the genes encoding σ factors and examined expression of the promoter for *yjbC* fused to *lacZ*. The typical cell growth in the sporulation medium with or without 2% glucose is shown (Supplementary Figure S2A). In the *sigX* and *sigM* mutants GI was reduced (**Figure 1**). In a double mutant of *sigX* and *sigM*, GI was completely lost, while in the *sigB* and *sigW* mutants, GI was still observed (**Figure 1**). Similar results were obtained using an *spx-lacZ* fusion (Supplementary Figure S3). This raised the possibility that *sigX* and s*igM* themselves might be induced by glucose.

### GI of *sigX* and *sigM* Expression

**FIGURE 2:**
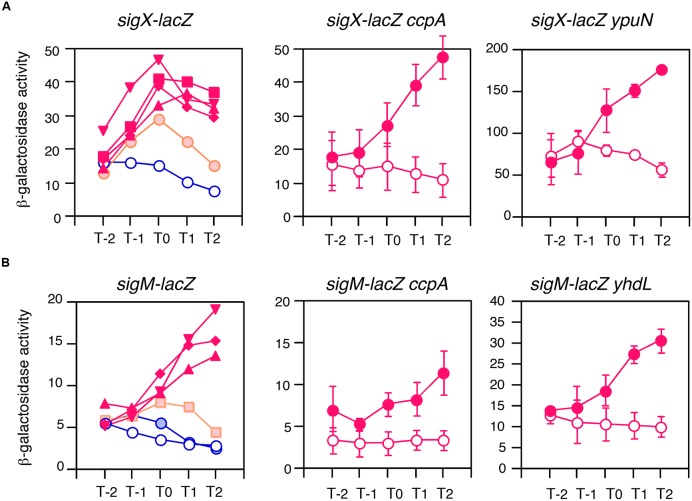
**Anti-σ factor-independent GI of *sigX* and *sigM*.** Cells were grown in sporulation medium and sampled hourly for β-galactosidase activities shown in Miller units. The X-axis is the same as that in **Figure 1**. Averages from at least three independent experiments are shown. **(A,B)** Show expression profiles of *sigX-lacZ* and *sigM-lacZ*, respectively. Each gene fusion is “Wt” in **Figure 4** and expressed only by their ECF σ-dependent promoters. Left panels in **(A,B)** show the concentrations of glucose-induced expression of the *lacZ* fusions. Graphs showing GI and absence of GI are shown in red and blue, respectively. Graphs showing mild GI are shown in orange. Open and closed symbols indicate no glucose and glucose-supplemented culture, respectively. Glucose concentrations: closed circles, 0.05%; squares, 0.1%; diamonds, 0.5%; triangles, 1%; reverse triangles, 2%. Standard deviations of all the points are less than 20%. In the middle and right panels in **(A,B)**, closed and open circles show expression with and without 2% glucose, respectively. Data sets showing GI are shown in red. Standard deviations (error bar) are shown. Middle panels show expression in the *ccpA* strain. Right panels show expression in the disruptant or depleted strain of anti-σ factor genes, *ypuN* for **(A)** and *yhdL* for **(B)**. The growth of the *yhdL* depleted strain is dependent on IPTG (*yhdL* expression is dependent on an IPTG-inducible promoter). Thus, pre-culture and culture for the expression test contained 1 mM and 0.05 mM IPTG, respectively.

To examine this possibility, we tested the expression of two genes in glucose-containing medium. As expected, *sigX* and *sigM* were induced by glucose in sporulation medium (**Figure 2**). This induction was very sensitive to low concentrations of glucose, 0.05% for *sigX* and 0.1% for *sigM*. In *B. subtilis* the *ccpA* gene has been known to play a major role in glucose-dependent gene induction and repression (Fujita, 2009). Thus, to test whether CcpA is involved in GI of *sig* genes, we examined the expression of *sigX* and *sigM* in a *ccpA* disruptant in the glucose-containing medium and observed normal GI (middle panels in **Figure 2**). This demonstrated that CcpA is not involved in GI.

The expression of *sigX* and *sigM* has been shown to be downregulated by their respective anti-σ factors (Brutsche and Braun, 1997; Horsburgh and Moir, 1999). To investigate the involvement of the anti-σ factors in the GI phenomenon regulating *sigX* and *sigM*, mutations in anti-σ factor genes were introduced into each strain with the *lacZ* fusion. It should be noted that a strain with an IPTG-dependent anti-σ^M^ factor gene was constructed and the expression was tested in medium containing 0.05 mM IPTG, since the anti-σ^M^ factor gene is essential (Horsburgh and Moir, 1999). We observed enhanced expression of both genes as expected, because the mutations increased the free forms of the σ factors. The increase was 3.5-fold for *sigX* and threefold for *sigM* (right panels in **Figure 2**). As shown in these panels, GI was observed in both mutants, indicating no involvement of anti-σ factors in GI of two σ-regulated genes. Among the seven ECF σ genes, only these two genes were subject to GI (Supplementary Figure S4). It should be noted that GI of *sigX* was observed in cells grown in LB medium (Supplementary Figure S5).

### GI of Sublancin Production

**FIGURE 3:**
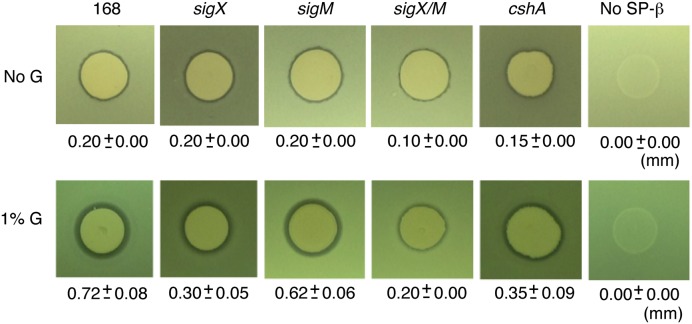
**Enhancement of sublancin production by glucose addition.** “1% G” and “No G” indicate the lawn cell plate supplemented with or without 1% glucose. The phenotypes of the strains are listed above the photographs. The strains were spotted onto Y13 lawn. The radii of the zones of inhibition produced by various strains were measured as the total diameter of the zone of clearing minus the diameter of the colony spot, divided by a factor of 2. Three independent experiments were performed and averages with standard deviations are shown. Strains; *sigX*, ASK4705; *sigM*, ASK4706; *sigX/M*, ASK4710; *cshA*, YDBRd; No SP-b, Y13.

*Bacillus subtilis* has been shown to produce Sublancin, an antimicrobial glycopeptide (Luo and Helmann, 2009). The precursor peptide-encoding gene and the self-resistance gene for Sublancin (*sunA* and *sunI*, respectively) are located on the SP-β prophage region in the chromosome (Luo and Helmann, 2009). The *sunA, sunT* (Sublancin transporter) and *sunS* (Sublancin glycosyltransferase) genes are activated by the transition regulator Abh, whose transcription requires σ^X^ in addition to σ^M^, although σ^M^ has a minor role (Luo and Helmann, 2009). Thus, GI of *sigX* might enhance Sublancin production through the σ^X^/Abh/SunA pathway. To test this possibility, we spotted Sublancin-producing wild type 168 cells on the lawn of Y13 cells lacking SP-β, which have defects in the production and immunity for Sublancin. On the 1% glucose-containing LB agar media, a larger inhibition zone of Y13 growth was observed as compared to that observed in LB medium (**Figure 3**). This indicates that glucose addition induces *sigX*, leading to enhanced transcription of *abh* and the operon containing *sunA*. Recently, glucose addition was shown to enhance Sublancin resistance in LB medium (Garcia De Gonzalo et al., 2015). Thus, the resistance levels of the lawn would be higher, and the apparent enhancement of inhibition zone would also be underestimated. As reported previously, we also observed a smaller inhibition zone in the *sigX* mutant (Luo and Helmann, 2009).

### The Minimum Promoter Region Required for GI

**FIGURE 4:**
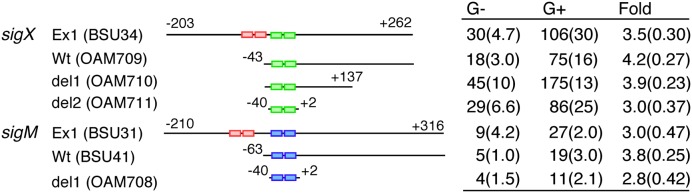
**Minimum promoter regions for GI.** Expression of various promoter *lacZ* transcriptional fusions is shown. The names of the *lacZ* fusion and the corresponding strain are shown. The pair of small rectangles on the horizontal line show the -35 and -10 elements of the promoter. Red, green and blue indicate σ^A^-, σ^X^-, and σ^M^-dependent promoters. The numbers indicate the nucleotide positions relative to the transcription start sites by ECF σ factors. The “G-” and “G+” columns show the β-galactosidase activities (Miller units) of each fusion grown in sporulation medium without or with 2% glucose, respectively. The activities were assayed at five time-points (log phase to early stationary phase), and the experiments were performed independently three times. Averages of the peak values with standard deviations are shown. “Fold” with calculated standard deviations through propagation of errors means extent of GI.

We determined a minimum promoter region responsible for GI in *sigX* and *sigM* by testing GI in *lacZ* fusions carrying various promoter regions. GI is expected to be observed in all constructs because the strains bear endogenous *sigX* or *sigM* genes. In both genes, similar levels of GI were observed in all fusions including the core promoter region fusions (-40 to +2 relative to the transcription start site, **Figure 4**). Moreover, between the core promoter regions of *sigX* and *sigM* there is no common nucleotide sequence except for the -35 and -10 elements for σ^X^-RNAP and σ^M^-RNAP. Thus, the similar levels of GI in all constructs suggested no involvement of some unknown trans-acting factors in GI.

### Dependency of GI of *sigX* and *sigM* on the Cellular Acetyl-CoA Pool

**FIGURE 5:**
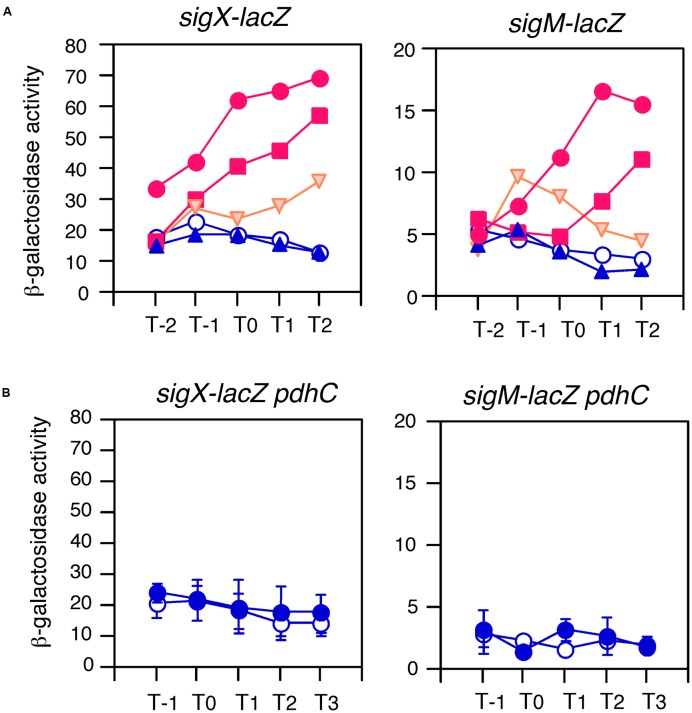
**Effects of various carbon sources and *pdhC* disruption on GI.** Cells were grown in sporulation medium and sampled hourly. β-galactosidase activities are shown in Miller units. The X-axis is the same as that in **Figure 1**. Averages from at least three independent experiments are shown. Each gene fusion (for *sigX-lacZ*, BSU43 and for *sigM-lacZ*, BSU41 are used) is indicated above the panels. **(A)** Graphs showing GI and absence of GI are shown in red and blue, respectively. Those showing mild GI are shown in orange. Open circles, control (no glucose); closed circles, 1% glucose; squares, 0.8% glycerol; reverse triangles, 0.8% pyruvate; triangles, 0.4% succinate. Standard deviations of all the points are less than 25%. **(B)** Data sets not showing GI are shown in blue. The same fusions were examined in the *pdhC* background in sporulation medium without (open symbols) or with (closed symbols) 2% glucose. Standard deviations (error bar) are shown.

We were interested in which carbon sources could induce *sigX* and *sigM* expression. Thus, three carbon sources were added, and we examined the expression levels of both *sig* genes. Glycerol or pyruvate addition induced gene expression to some extent, while succinate did not (**Figure 5A**). We note that the effects of pyruvate were modest. Glycerol and pyruvate have been shown to be incorporated into the glycolysis pathway and succinate is not (Wang et al., 2010). Thus, this pattern is reminiscent of acetyl-CoA production. Recently, protein-lysine acetylation has been studied in *B. subtilis* (Kim et al., 2013; Kosono et al., 2015; Carabetta et al., 2016). This modification may change protein function. In *Escherichia coli*, glucose addition changed and enhanced global acetylation profiles (Schilling et al., 2015). Thus, we hypothesized that glucose addition might result in a higher cellular pool of acetyl-CoA, leading to enhancement of acetylation of proteins regulating *sigX* and *sigM*. To examine the involvement of acetyl-CoA in GI of *sigX* and *sigM*, a disruption mutation of *pdhC*, which encodes the E2 subunit of pyruvate dehydrogenase and whose reaction product is acetyl-CoA (Gao et al., 2002), was introduced into the *lacZ* fusion strain. The rationale for the experiment is that in the *pdhC* mutant an acetyl-CoA pool would be reduced, although the cells still have other acetyl-CoA synthesis pathway involving acetyl-CoA synthetase. It should be noted that the gene encoding acetyl-CoA synthetase has been known to be repressed by glucose (Grundy et al., 1994). In the resultant strains, GI of *sigX* and *sigM* was completely abolished (**Figure 5B**). We observed slow growth and poor cell mass of the mutants as previously reported (Gao et al., 2002, Supplementary Figure S2B). These results strongly suggested acetyl-CoA dependent GI of *sigX* and *sigM*.

### Identification of *cshA* as a Positive Regulator by Transposon Mutagenesis

To further gain insights into the mechanism of GI, we performed transposon mutagenesis for GI-deficient mutants by using a transposon delivery vector, pMarA (Le Breton et al., 2006). Mutants with *sigX-lacZ* showing pale blue on LB or sporulation agar plate containing X-Gal and 2% glucose were chosen among about 12,000 colonies. Several mutants were obtained and one was a disruptant of *cshA*, which encodes DEAD-box RNA helicase (Lehnik-Habrink et al., 2010). Transposon was inserted into the 342nd codon of the *cshA* ORF. In this mutant, *sigX* and *sigM* expression was not induced by glucose and basal expression was reduced but not abolished (Supplementary Figure S6). The decrease in *sigX* expression resulted in a decrease in Sublancin production in the *cshA* mutant (**Figure 3**). Among many candidates of the Tn mutagenesis, only additional four mutations negatively affected GI of both *sigX* and *sigM* genes (data not shown). Two carried a disruption of a gene of which function was not yet identified (*ylxR* and *yqfO*) and the rests were *ptsH* and *gcp* mutants. Probably in the *ptsH* mutant efficient glucose transport would be inhibited, leading to abolishment of GI. The detailed analysis of these mutants will be reported elsewhere.

### Operon Structure and Expression of *cshA*

The chromosomal region surrounding *cshA* is shown in Supplementary Figure S7A, *cshA* seems to be a monocistronic operon with its own promoter and terminator (Nicolas et al., 2012). Thus, we created a promoter-*lacZ* fusion of *cshA* and examined its expression. The expression was observed and found to be unaffected by glucose addition (Supplementary Figure S7B, left). The σ^X/M/V^-driven promoter within the *ydbO* ORF has been reported to co-transcribe *ydbO-(ydbP)-ddlA-murF* (Eiamphungporn and Helmann, 2008). Between *murF* and *cshA* there is no terminator, and thus this promoter may transcribe *cshA*. In addition, there is a promoter upstream of *ddlA* (Nicolas et al., 2012). These suggested a read-through mechanism from the upstream promoters. To confirm this, we examined activities of a *lacZ*-fusion located at the original locus in the chromosome. This *lacZ* fusion showed higher activity as compared to its own promoter fused to *lacZ*. Glucose addition also did not affect the activity (Supplementary Figure S7B, right). It should be noted that the strain with this fusion at the original locus carries the *cshA* disruption. These observations showed that read-through transcription contributed to *cshA* expression. It was previously reported that the *cshA* expression is constitutive at the protein level (Lehnik-Habrink et al., 2010).

### Complementation of the *cshA* Mutation by *cshA* in Trans

**FIGURE 6:**
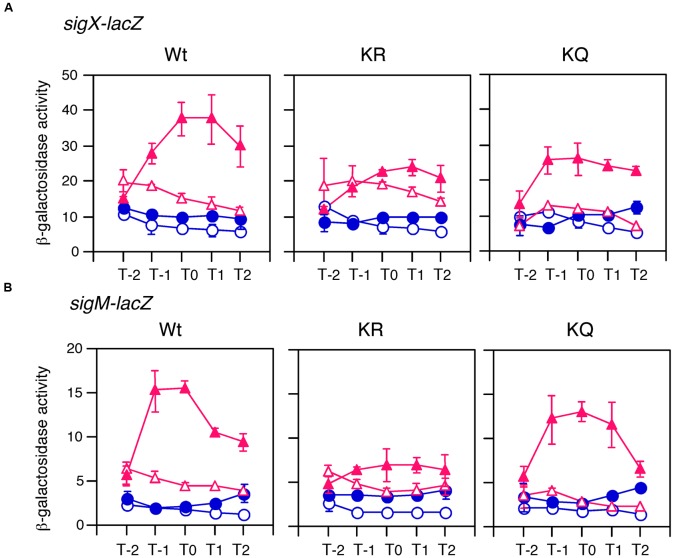
**Complementation test of *cshA* for GI and the effects of substitutions of acetylation residues in CshA on GI of *sigX* and *sigM*.** Cells were grown in sporulation medium with (closed symbols) or without (open symbols) 1 and 0.5% glucose for **(A,B)**, respectively. Triangles and circles indicate the culture with or without 2% xylose, respectively. Cells were sampled hourly and β-galactosidase activities are shown in Miller units. The X-axis is the same as that in **Figure 1**. Data sets with and without xylose are shown in red and blue, respectively. Averages from at least three independent experiments and standard deviations (error bar) are shown. Structures for the xylose-inducible *cshA* cassette are shown in Supplementary Figure S1. “Wt,” “KR,” and “KQ” mean strains with the genes encoding wild type CshA, CshA with two K to R substitutions, and CshA with two K to Q substitutions, respectively. **(A)** For Wt, KR, and KQ, OAM725, OAM727, and OAM729 were used. **(B)** For Wt, KR, and KQ, OAM724, OAM726, and OAM728 were used.

We created a xylose-inducible *cshA* cassette at the *bkdB* locus in the *cshA* mutant with *sigX-lacZ* or *sigM-lacZ* (Supplementary Figure S1A). Xylose addition to the culture of these strains without glucose recovered the basal expression levels of *sigX-lacZ* and *sigM-lacZ* (left panels in **Figure 6**). Xylose addition to the culture with glucose also rescued GI of these strains, demonstrating that the *cshA* gene is responsible for GI. We noted that xylose addition did not have any effect on GI in the control strain with *sigX-lacZ* nor *sigM-lacZ* (data not shown).

### Stimulation of the Acetylation of CshA by Glucose Addition

**FIGURE 7:**
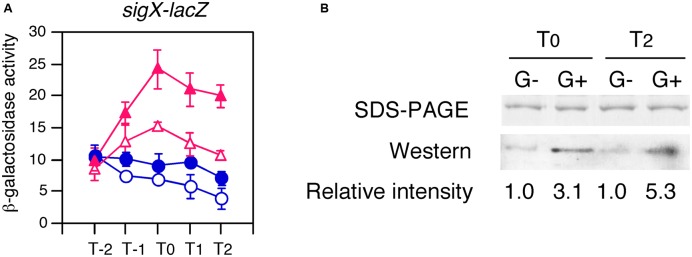
**Enhancement of acetylation of CshA by glucose addition. (A)** Confirmation that His-CshA is functional. OAM731 cells with the xylose-inducible gene encoding His-tagged CshA were grown in sporulation medium with (closed symbols) or without (open symbols) 1% glucose. Triangles and circles indicate the culture with or without 2% xylose, respectively. Cells were sampled hourly. β-galactosidase activities are shown in Miller units. The X-axis is the same as that in **Figure 1**. Data sets with and without xylose are shown in red and blue, respectively. Averages from at least three independent experiments and standard deviations (error bar) are shown. **(B)** Western blot using anti-acetylated lysine residue antibody. OAM730 cells were grown in 800 ml sporulation medium containing 2% xylose with or without 1% glucose and harvested at T0 and T2. Each His-CshA was purified using Ni-affinity column, analyzed using 15% SDS-PAGE, and stained with Coomassie blue (top panel). Equal amount of the protein (0.1 μg) was electrophoresed in 15% SDS-PAGE and blotted onto a nitrocellulose membrane. Western blot analysis with monoclonal anti-acetylated lysine antibody is shown in the bottom panel. The “G-” and “G+” show the protein purified from the culture without or with 1% glucose, respectively.

We added His-tag to the CshA protein encoded by the cassette at *bkdB* using PCR (Supplementary Figure S1B). The His-CshA protein was confirmed to be functional by a complementation test (**Figure 7A**). Xylose addition to the culture with or without glucose caused expression of His-CshA, and the two protein fractions containing His-CshA from the two time points (T0 and T2), were purified by Ni-affinity column (**Figure 7B**). A global analysis of the acetylome in *B. subtilis* revealed that CshA was acetylated at K244 and K296 (Kosono et al., 2015). Thus, we examined the acetylation status of purified His-CshA by Western blot analysis using an anti-acetyl lysine antibody. The acetyl lysine signals were enhanced in His-CshA purified from the cells with glucose, indicating that glucose addition stimulates CshA acetylation (**Figure 7B**). A similar result was reproducibly observed from independent culture (data not shown).

### GI of *sigX* and *sigM* in the *rny* Mutant

**FIGURE 8:**
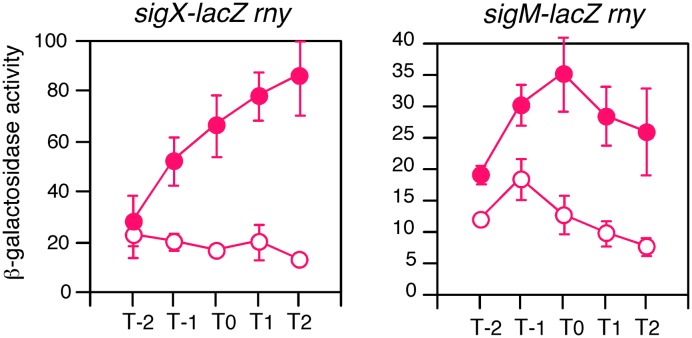
**GI in the RNase Y-depleted mutant.** Cells were grown in sporulation medium with (closed symbols) or without (open symbols) 2% glucose. Left, *sigX-lacZ* with *rny* (OAM735); right, *sigM-lacZ* with *rny* (OAM734). Cells were sampled hourly and β-galactosidase activities are shown in Miller units. The X-axis is the same as that in **Figure 1**. Data sets showing GI are shown in red. Averages from at least three independent experiments and standard deviations (error bar) are shown. The mutant of *rny* disruption is associated with Pxyl -*rny*, and thus the strains were maintained in the presence of 1% xylose and the preculture for the experiments lacked xylose.

CshA has been known to be involved in RNA degradosome containing endoribonuclease RNase Y (Lehnik-Habrink et al., 2010). RNase Y has global impact on the gene expression through mRNA metabolism (Lehnik-Habrink et al., 2011; Durand et al., 2012; Laalami et al., 2013). Thus, CshA may cause GI of *sigX* and *sigM* due to the inhibition of RNase Y activity through acetylated CshA-dependent mechanism, leading to the longer half-life of mRNAs of *sigX* and *sigM*. Otherwise RNase Y may regulate *sigX* and *sigM* indirectly. Indeed, *sigM* expression was enhanced in an RNase Y-depleted mutant, *rny*, according to the three analyses (Lehnik-Habrink et al., 2011; Durand et al., 2012; Laalami et al., 2013). To examine this, we introduced *rny* depletion into the strains bearing *sigX-lacZ* or *sigM-lacZ* and tested whether GI was observed. GI of *sigX* was clearly observed in the *rny* mutant (**Figure 8**, left). With respect to *sigM*, we observed enhancement of *sigM-lacZ* expression especially in the log phase as expected (**Figure 8**, right). Moreover, GI of *sigM* was also clearly observed. These results demonstrated that CshA would act on expression of *sigX* and *sigM* independent of influence to RNase Y due to interaction of CshA with RNA degradosome.

### The Effects of K–R and K–Q Mutations on GI

K–R and K–Q mutations have been shown to mimic non-acetylation and acetylation states, respectively (Kamieniarz and Schneider, 2009). Hence, we also introduced these mutations into the xylose-inducible *cshA* cassette system (Supplementary Figure S1A). Introduction of these mutations resulted in similar effects on the expression of *sigX-lacZ* and *sigM-lacZ* (**Figure 6**). In the strain with CshA bearing two K–R substitutions, basal expression was recovered to that of wild type CshA, whereas GI was completely abolished. In the strain with CshA bearing two K–Q substitutions, basal expression was not recovered, whereas GI was observed. These results strongly suggested that acetylation of two lysine residues in CshA is required for GI of *sigX* and *sigM*. Moreover, the results also indicated that basal expression and GI of *sigX* and *sigM* were mutually distinguishable in a genetic context. We note that the strains bearing wild type and mutant CshA proteins did not grow in culture with 2% glucose in the presence of xylose, and the extent of the resistance against glucose changed in the strains bearing *sigX-lacZ* and *sigM-lacZ* due to unknown reasons (data not shown). Thus, different glucose concentrations were used for the experiments for *sigX-lacZ* and *sigM-lacZ.*

**FIGURE 9:**
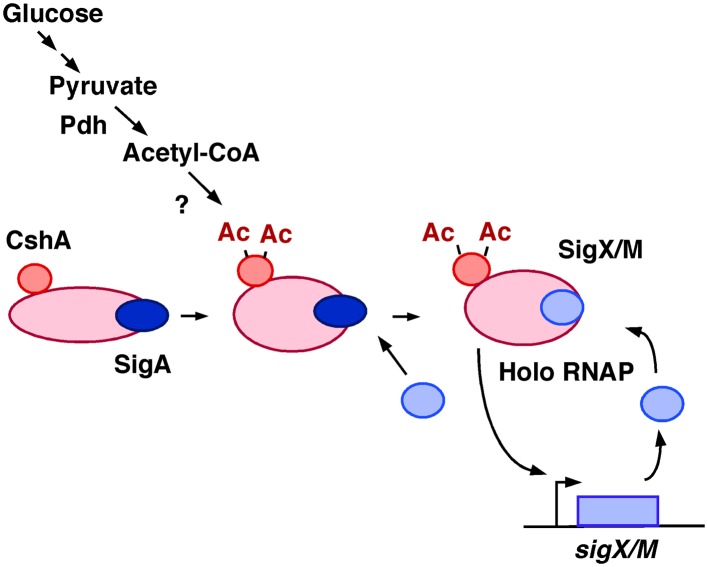
**Model of GI of *sigX* and *sigM*.** Glucose addition would result in an increase of the cellular acetyl-CoA pool, leading to more acetylated CshA. CshA has been shown to associate with RNAP. RNAP with acetylated CshA may stimulate replacement of σ^X/M^ for σ^A^ in the RNAP holoenzyme, although the mode of action is not known. Moreover, the acetyl transfer enzyme that acetylates CshA is also not known. Ac, acetyl moiety; Pdh, pyruvate dehydrogenase.

Recent analysis using mass spectrometry of *B. subtilis* RNAP reported that half of the RNAP pool was associated with CshA (Delumeau et al., 2011). Thus, we present a model for the mechanism by which GI of *sigX and sigM* occurs as shown in **Figure 9**.

## Discussion

Extracytoplasmic function σ factor genes have been shown to be induced mainly by cell envelope and cell membrane stresses through a mechanism involving anti-σ factor embedded in the cell membrane. As an exception, *Caulobacter crescentus* ECF σ factors play roles in many other stresses (for example, heat, glucose starvation, and oxidative stresses), because stress-responsive sigma factor homologs of σ^B^ in *B. subtilis* and σ^S^ in *E. coli* are lacking in *C. crescentus*. Metabolic stress such as carbon-starvation induced ECF σ factor σ^T^ perhaps in an anti-σ factor dependent manner in this bacterium (Britos et al., 2011). Our study finds that glucose addition to the medium induces the *sigX* and *sigM* genes independent of their anti-σ factors, probably through a CshA-dependent functional modification of RNAP. This is the first report for GI of ECF σ genes to our knowledge. The addition of glucose to the medium affects σ^X^- and σ^M^-dependent cellular functions, such as Sublancin production and Spx overproduction.

RNA polymerase with acetylated CshA may somehow stimulate the autoregulatory loop of *sigX* and *sigM*. Our model shown in **Figure 9** encompasses all of the experimental data: involvement of *pdhC*, the different effects of carbon sources on GI, the effects of non-acetylation mimicking mutations, and the enhancement of CshA acetylation by glucose, yet some key elements in this model remain unknown. It is of interest whether CshA is acetylated before it binds to RNAP or if acetylation of CshA stimulates RNAP-binding of CshA. In addition, there are several outstanding questions, for example, what enzyme acetylates CshA, or how σ^X/M^ replaces σ^A^ in the RNAP holoenzyme, during GI. According to an analysis of the RNAP holoenzyme, the core RNAP with no σ factor was barely detected (Delumeau et al., 2011). Thus, replacement of σ^X/M^ for σ^A^ is a likely event. Generally, competition between housekeeping σ^A^ and alternative σ factors for the core RNAP is favorable for σ^A^-binding due to the higher affinity of σ^A^ for RNAP (Österberg et al., 2011). To change the balance of σ binding, various mechanisms have been reported (Österberg et al., 2011). For example, the small subunit of RNAP, the ω- subunit, is involved in σ factor recruitment (Weiss and Shaw, 2015). This demonstrates that a protein associated with RNAP can modulate its affinity to a specific σ factor perhaps through structural change as we may see with CshA.

Thousands of protein acetylation sites have been reported in *B. subtilis*, but with respect to transcription factors, a link between acetylation and a functional change has only been elucidated in very rare cases (Thao et al., 2010; Lima et al., 2011; Hu et al., 2013; Hentchel et al., 2015). Generally, the link to function was shown by introducing acetylation-mimicking and -abolishing mutations into the protein. In our study, the functional modulation of CshA by acetylation was also clarified using this method.

CshA has been characterized as a multi-functional protein and required for growth in low temperatures (Lehnik-Habrink et al., 2013). CshA exists in an RNA degradosome complex and affects the expression of many genes through its interaction with RNase Y (Lehnik-Habrink et al., 2013). In the case of *sigX* and *sigM*, GI was observed in *rny*-depleted mutant. This indicates that the mechanism of CshA-dependent GI does not occur via mRNA metabolism by RNase Y. Moreover, CshA is required for ribosome biogenesis, and thus CshA has a role in translation (Lehnik-Habrink et al., 2013). A possibility that translational control of *sigX* and *sigM* by CshA cannot be completely excluded. However, it is difficult to speculate how the changes in ribosome biogenesis caused by the *cshA* depletion results in the specific regulation of *sigX* and *sigM* expression. Thus, we favored the model shown in **Figure 9**, although further experiments will be required to prove the model.

## Author Contributions

MO and KA designed research, performed experiments and analyzed results. MO wrote the paper.

## Conflict of Interest Statement

The authors declare that the research was conducted in the absence of any commercial or financial relationships that could be construed as a potential conflict of interest.
