# Supplementary material for: Glucose Induces ECF Sigma Factor Genes, sigX and sigM, Independent of Cognate Anti-sigma Factors through Acetylation of CshA in Bacillus subtilis
Source: Front Microbiol. 2016 Nov 29;7:1918. doi: 10.3389/fmicb.2016.01918 (PMC5126115; doi:10.3389/fmicb.2016.01918)
Supplement: Supplementary file 1 [file Data_Sheet_1.PDF]

Supplementary References

Asai, K., Ishiwata, K., Matsuzaki, K., and Sadaie, Y. (2008). A viable *Bacillus subtilis* strain without functional extracytoplasmic function sigma genes. *J. Bacteriol.* 190, 2633-2636.

Asai, K., Yamaguchi, H., Kang, C. M., Yoshida, K., Fujita, Y., and Sadaie ,Y. (2003). DNA microarray analysis of *Bacillus subtilis* sigma factors of extracytoplasmic function family. *FEMS Microbiol. Lett*. 220, 155-160.

Boylan, S. A., Thomas, M.D., and Price, C.W. (1991). Genetic method to identify regulons controlled by nonessential elements: isolation of a gene dependent on alternate transcription factor sigma B of *Bacillus subtilis*. *J. Bacteriol.* 173, 7856-7866.

Gao, H., Jiang, X., Pogliano, K., and Aronson, A. I. (2002). The E1beta and E2 subunits of the *Bacillus subtilis* pyruvate dehydrogenase complex are involved in regulation of sporulation. *J. Bacteriol.* 184, 2780-2788.

Hori, K., Kaneko, M., Tanji, Y., Xing, X. H., and Unno, H. (2002). Construction of self-disruptive *Bacillus megaterium* in response to substrate exhaustion for polyhydroxybutyrate production. *Appl. Microbiol. Biotechnol.* 59, 211-216.

Ishii, H., Tanaka, T., and Ogura, M. (2013). The *Bacillus subtilis* response regulator gene *degU* is positively regulated by CcpA and by catabolite-repressed synthesis of ClpC. *J. Bacteriol*. 195, 193-201.

Itaya, M. (1992). Construction of a novel tetracycline resistance gene cassette useful as a marker on the *Bacillus subtilis* chromosome. *Biosci. Biotechnol. Biochem.* 56, 685-686.

Kosono, S., Asai, K., Sadaie, Y., and Kudo, T. (2004). Altered gene expression in the transition phase by disruption of a Na+/H+ antiporter gene (*shaA*) in *Bacillus subtilis*. *FEMS Microbiol. Lett*. 232, 93-99.

Le Breton, Y., Mohapatra, N. P., and Haldenwang, W. G. (2006). In vivo random mutagenesis of *Bacillus subtilis* by use of TnYLB-1, a mariner-based transposon. *Appl. Environ. Microbiol.* 72, 327-333.

Lehnik-Habrink, M., Schaffer, M., Mäder, U., Diethmaier, C., Herzberg, C., and Stülke, J. (2011). RNA processing in *Bacillus subtilis*: identification of targets of the essential RNase Y. *Mol. Microbiol.* 81, 1459-1473.

Ogura, M. (2016). Post-transcriptionally generated cell heterogeneity regulates biofilm formation in *Bacillus subtilis. Genes Cells* 21, 335-349.

Ogura, M., and Tsukahara, K. (2010). Autoregulation of the *Bacillus subtilis* response regulator gene *degU* is coupled with the proteolysis of DegU-P by ClpCP. *Mol. Microbiol.* 75, 1244-1259.

Ogura, M., Shimane, K., Asai, K., Ogasawara, N., and Tanaka, T. (2003). Binding of response regulator DegU to the *aprE* promoter is inhibited by RapG, which is counteracted by extracellular PhrG in *Bacillus subtilis*. *Mol. Microbiol.* 49, 1685-1697.

Shiwa, Y., Yoshikawa, H., Tanaka, T., and Ogura, M. (2015). *Bacillus subtilis degSU* operon is regulated by the ClpXP-Spx regulated proteolysis system. *J. Biochem.* 157, 321-330.

Steinmetz, M., and Richter, R. (1994). Plasmids designed to alter the antibiotic resistance expressed by insertion mutations in *Bacillus subtilis,* through in vivo recombination. *Gene* 142, 79-83.

Table S1. Strains and plasmids used in the study.

Strain	Genotype	Reference or source
168	*trpC2*	Laboratory stock
PB153	*trpC2 sigB* (Cm^{r})	Boylan et al., 1991
ASK2001	*trpC2 sigM* (Cm^{r})	Asai et al., 2008, K. Kobayashi
ASK2004	*trpC2 sigX* (Cm^{r})	Asai et al., 2008, K. Kobayashi
ASK4711	*trpC2 sigX* (Cm^{r}::Sp^{r})	This study
ASK2003	*trpC2 sigW* (Cm^{r})	Asai et al., 2008, K. Kobayashi
ASK4712	*trpC2 sigW* (Cm^{r}::Km^{r})	This study
ASK4705	*trpC2 sigX*	Asai et al., 2008
ASK4706	*trpC2 sigM*	Asai et al., 2008
ASK4710	*trpC2 sigX sigM*	Asai et al., 2008
YDBRd	*trpC2 ydbR-lacZ* (Em^{r}) *ydbR (cshA)*	BSORF
Y13 (MGB12)	*pro7 pro5*	US patent US7981659 B2
BSF2842	*trpC2 spx-lacZ* (Em^{r}) *spx*	BSORF
OAM698	*trpC2 spx-lacZ* (Em^{r}) *spx sigB* (Cm^{r})	This study
OAM699	*trpC2 spx-lacZ* (Em^{r}) *spx sigM* (Cm^{r})	This study
OAM700	*trpC2 spx-lacZ* (Em^{r}) *spx sigX* (Cm^{r})	This study
OAM701	*trpC2 spx-lacZ* (Em^{r}) *spx sigW* (Km^{r})	This study
OAM692	*trpC2 amyE*::*yjbC-lacZ* (Cm^{r})	Shiwa et al., 2015
OAM702	*trpC2 amyE*::*yjbC-lacZ* (-172 to +21 relative to the translation start site, Cm^{r}::Tc^{r})	This study
OAM703	*trpC2 amyE*::*yjbC-lacZ* (Cm^{r}::Tc^{r}) *sigB* (Cm^{r})	This study
OAM704	*trpC2 amyE*::*yjbC-lacZ* Cm^{r}::Tc^{r}) *sigM* (Cm^{r})	This study
OAM705	*trpC2 amyE*::*yjbC-lacZ* (Cm^{r}::Tc^{r}) *sigX* (Cm^{r})	This study
OAM706	*trpC2 amyE*::*yjbC-lacZ* (Cm^{r}::Tc^{r}) *sigW* (Km^{r})	This study
OAM707	*trpC2 amyE*::*yjbC-lacZ* (Cm^{r}::Tc^{r}) *sigM* (Cm^{r}) *sigX* (Sp^{r})	This study
BSU31	*trpC2 amyE*::*sigM-lacZ* Ex1 (-210 to +316, Cm^{r})	Asai et al., 2003
BSU41	*trpC2 amyE*::*sigM-lacZ* Wt (-63 to +316, Cm^{r})	Kosono et al., 2004
OAM708	*trpC2 amyE*::*sigM-lacZ* del1 (-40 to +2, Cm^{r})	This study
BSU34	*trpC2 amyE*::*sigX-lacZ* Ex1 (-203 to +262, Cm^{r})	Asai et al., 2003
BSU43	*trpC2 amyE*::*sigX-lacZ* Wt (-43 to +262, Cm^{r})	Kosono et al., 2004
OAM709	*trpC2 thrC*::*sigX-lacZ* Wt (-43 to +262, Em^{r})	This study
OAM710	*trpC2 amyE*::*sigX-lacZ* del1 (-43 to +137, Cm^{r})	This study
OAM711	*trpC2 thrC*::*sigX-lacZ* del2 (-40 to +2, Em^{r})	This study
BSU32	*trpC2 amyE*::*sigV-lacZ* (Cm^{r})	Asai et al., 2003
BSU33	*trpC2 amyE*::*sigW-lacZ* (Cm^{r})	Asai et al., 2003
BSU35	*trpC2 amyE*::*sigY-lacZ* (Cm^{r})	Asai et al., 2003
BSU36	*trpC2 amyE*::*sigZ-lacZ* (Cm^{r})	Asai et al., 2003
BSU37	*trpC2 amyE*::*ylaC-lacZ* (Cm^{r})	Asai et al., 2003
TF10	*trpC2 ccpA* (Cm^{r})	Ishii et al., 2013, K. Kobayashi
501-77	*trpC2 pheA1 pdhC* (Km^{r})	Gao et al., 2002
YhdLp	*trpC2 yhdL* (Em^{r}, *yhdL-lacZ*)	BSORF
OAM712	*trpC2 yhdL* (Em^{r}, *lacZ*::Tc^{r})	This study
ASK443	*trpC2 ypuN* (Em^{r})	This study
GP193	*rny* (Pxyl-*rny*, Cm^{r})	Lehnik-Habrink et al., 2011
OAM714	*trpC2 amyE*::*sigM-lacZ* Wt (-63 to +316, Cm^{r}) *yhdL* (Em^{r}, *lacZ*::Tc^{r})	This study
OAM715	*trpC2 amyE*::*sigM-lacZ* Wt (-63 to +316, Cm^{r}::Tc^{r}) *ccpA* (Cm^{r})	This study
OAM716	*trpC2 amyE*::*sigM-lacZ* Wt (-63 to +316, Cm^{r}) *pdhC* (Km^{r})	This study
OAM734	*trpC2 amyE*::*sigM-lacZ* Wt (-63 to +316, Cm^{r}::Tc^{r}) *rny* (Pxyl-*rny*, Cm^{r})	This study
OAM717	*trpC2 amyE*::*sigX-lacZ* Wt (-43 to +262, Cm^{r}) *ypuN* (Em^{r})	This study
OAM718	*trpC2 amyE*::*sigX-lacZ* Wt (-43 to +262, Cm^{r}::Tc^{r}) *ccpA* (Cm^{r})	This study
OAM719	*trpC2 amyE*::*sigX-lacZ* Wt (-43 to +262, Cm^{r}::Tc^{r}) *pdhC* (Km^{r})	This study
OAM735	*trpC2 amyE*::*sigX-lacZ* Wt (-43 to +262, Cm^{r}::Tc^{r}) *rny* (Pxyl-*rny*, Cm^{r})	This study
OAM720	*trpC2 amyE*::*sigM-lacZ* Wt (-63 to +316, Cm^{r}) *cshA*::Tn (Km^{r})	This study
Tn I-X	*trpC2 thrC*::*sigX-lacZ* Wt (-43 to +262, Em^{r}) *cshA*::Tn (342th codon, Km^{r})	This study
Tn I-M	*trpC2 amyE*::*sigM-lacZ* Wt (-63 to +316, Cm^{r}) *cshA*::Tn (342th codon, Km^{r})	This study
OAM722	*trpC2 cshA* (Tc^{r})	This study
OAM-N70	*bkdB*::*sinR* (Km^{r})	Ogura, 2016
OAM732	*trpC2 thrC*::P*cshA-lacZ* (Em^{r})	This study
OAM733	*trpC2 thrC*::P*cshA-lacZ* (Em^{r}) *cshA* (Tc^{r})	This study
OAM723	*trpC2 bkdB*::*xylR*-Px-*cshA* (Km^{r})	This study
OAM724	*trpC2 amyE*::*sigM-lacZ* Wt (-63 to +316, Cm^{r}) *cshA* (Tc^{r}) *bkdB*::*xylR*-Px-*cshA* (Km^{r})	This study
OAM725	*trpC2 thrC*::*sigX-lacZ* Wt (-43 to +262, Em^{r}) *cshA* (Tc^{r}) *bkdB*::*xylR*-Px-*cshA* (Km^{r})	This study
OAM726	*trpC2 amyE*::*sigM-lacZ* Wt (-63 to +316, Cm^{r}) *cshA* (Tc^{r}) *bkdB*::*xylR*-Px-*cshA* (K244R, K296R, Km^{r})	This study
OAM727	*trpC2 thrC*::*sigX-lacZ* Wt (-43 to +262, Em^{r}) *cshA* (Tc^{r}) *bkdB*::*xylR*-Px-*cshA* (K244R, K296R, Km^{r})	This study
OAM728	*trpC2 amyE*::*sigM-lacZ* Wt (-63 to +316, Cm^{r}) *cshA* (Tc^{r}) *bkdB*::*xylR*-Px-*cshA* (K244Q, K296Q, Km^{r})	This study
OAM729	*trpC2 thrC*::*sigX-lacZ* Wt (-43 to +262, Em^{r}) *cshA* (Tc^{r}) *bkdB*::*xylR*-Px-*cshA* (K244Q, K296Q, Km^{r})	This study
OAM730	*trpC2 cshA* (Tc^{r}) *bkdB*::*xylR*-Px-*cshA*-Hisx6 (Km^{r})	This study
OAM731	*trpC2 thrC*::*sigX-lacZ* Wt (-43 to +262, Em^{r}) *cshA* (Tc^{r}) *bkdB*::*xylR*-Px-*cshA*-Hisx6 (Km^{r})	This study

Plasmid	Description	Reference or source
pIS284	Amp^{r} *amyE*::*lacZ* Cm^{r}	Tsukahara and Ogura,2008, I Smith
pIS284-sigM-del1	Amp^{r} *amyE*::*sigM-lacZ* (-40 to +2) Cm^{r}	This study
pDG1663	Amp^{r} Sp^{r} *thrC*::*lacZ* Em^{r}	Guerout-Fleury et al., 1996
pDG1663-sigX-Wt	Amp^{r} Sp^{r} *thrC*::*sigX-lacZ* (-43 to +262) Em^{r}	This study
pDG1663-sigX-del2	Amp^{r} Sp^{r} *thrC*::sigX-*lacZ* (-40 to +2) Em^{r}	This study
pDL2	Amp^{r} *amyE*::*lacZ* Cm^{r}	Yuan and Wong, 1995
pDL2-sigX-del1	Amp^{r} *amyE*::*sigX-lacZ* (-43 to +137) Cm^{r}	This study
pMarA	Amp^{r} Em^{r} Km^{r}	Le Breton et al., 2006
pDG1729	Amp^{r} Em^{r} *thrC*::*lacZ* Sp^{r}	Guerout-Fleury et al., 1996
pDG1729-PcshA	Amp^{r} Em^{r} *thrC*::PcshA-*lacZ* (+361 to -1 relative to the translation start site) Sp^{r}	This study
pX	Amp^{r} *amyE*::*xylR*-Pxyl Cm^{r}	Hori et al., 2002
pBEST304	Amp^{r} Tc^{r}	Itaya, 1992
pLacZ::Tc	Amp^{r} *lacZ*::Tc^{r}	Ogura et al., 2003
ECE75	Amp^{r} Cm^{r}::Tc^{r}	Steinmetz and Richter, 1994
pMUTIN3DZ	Amp^{r} Em^{r} Pspac	Yoshimura et al., 2004
pMUTIN3DZ-ypuN	Amp^{r} Em^{r} Pspac carrying a part of *ypuN*	This study

With respect to *sigM* and *sigX*, the cloning promoter regions are refferred to relative to the transcription start sites by SigM-RNAP and SigX-RNAP, respectively.

Table S2. Oligonucleotides used for this study.

Name	Sequence	Product/use
PsigM-F	5'-AATTAATGTGCAACTTTAAACCTTTCTTATGCGTGTATAACATAG-3'	pIS284-sigM-del1
PsigM-R	5'-GATCCTATGTTATACACGCATAAGAAAGGTTTAAAGTTGCACATT-3'	pIS284-sigM-del1
SigX-R	5'-CATGGATCCGAGTATGCGCTGTCTG-3'	pDG1663-sigX-Wt
SigX-F	5'-CATGAATTCAGTTGTAATGTAACTTTTCAAGC-3'	pDG1663-sigX-Wt, pDL2-sigX-del1, EMSA probe sigX
SigX-R2	5'-CATGGATCCGGATATAAACCTCTTGAAGAAG-3'	pDL2-sigX-del1
PsigX-F	5'-AATTGTAATGTAACTTTTCAAGCTATTCATACGACAAAAAAGTGA-3'	pDG1663-sigX-del2
PsigX-R	5'-GATCTCACTTTTTTGTCGTATGAATAGCTTGAAAAGTTACATTAC-3'	pDG1663-sigX-del2
pX-cshA-F(Gib)	5'-TGACAAATGGTCCAAATTGTCTAAAGGGCAGTTTTATAA-3'	Pair 1, 6
cshA-FRm	5'-CGAATTTTCTACGTTCTTGCACTTCC-3'	Pair 1 for KR
cshA-Fm	5'-GTGCAAGAACGTAGAAAATTCG-3'	Pair 2 for KR
cshA-Rm	5'-CGCAACCATACGTCTCGCCT-3'	Pair 2 for KR
cshA-RFm	5'-AGGCGAGACGTATGGTTGCGCTT-3'	Pair 3 for KR
cshA-FRm2	5'-CGAATTTTTGACGTTCTTGCACTTCC-3'	Pair 1 for KQ
cshA-F-KQ	5'GTGCAAGAACGTCAAAAATTCG--3'	Pair 2 for KQ
cshA-R-KQ	5'-CGCAACCATACGTTGCGCCT-3'	Pair 2 for KQ
cshA-RFm2	5'-AGGCGCAACGTATGGTTGCGCTT-3'	Pair 3 for KQ
cshA-R(Gib)	5'-ATGCTATTCATTTGCGGGTTAGTAAGATTTTTTCTGGCGTCT-3'	Pair 3, 6
bkdB-FF	5'-ATCGGCAAGGCGGACGTAAAAAGGG-3'	Pair 4, 8, 9
Km-F	5'-GACACAAGCATGACCATTATGA-3'	Pair 4
xylR-F(Km)	5'-AATGGTCATGCTTGTGTCAACTAACTTATAGGGGTAACACT-3'	Pair 5
pxyl-R	5'-TTTGGACCATTTGTCATTTCCCC-3'	Pair 5
bkdB-RF	5'-CCCGCAAATGAATAGCATGTGG-3'	Pair 7
bkdB-RR	5'-CATCAATTACGCCGGATATGG-3'	Pair 7, 8
cshA-His-R(Gib)	5'-ATGCTATTCATTTGCGGGTTAGTGATGGTGATGGTGGTGGTAAGATTTTTTCTGGCGTC-3'	Pair 9
cshA-FR(Tc)	5'-GCTGTTCATATCGACCCGAATTTTTTACGTTCTTGCACTTCC-3'	cshA (Tc^r)
cshA-RF(Tc)	5'-TTTTTTTATAACAGGAATTCAGGCGAAACGTATGGTTGCGCTT-3'	cshA (Tc^r)
cshA-FF	5'-CGCGATACGAACATATTCGTCCGG-3'	cshA (Tc^r)
cshA-RR	5'-GAAGAGCGCTGGTTCTTGTA-3'	cshA (Tc^r)
TC-F	5'-GGTCGATATGAACAGCTTATTTAC-3'	cshA (Tc^r)
TC-R	5'-GAATTCCTGTTATAAAAAAAGGATCAA-3'	cshA (Tc^r)
pDG1729-cshA-E	5'-ATTGAATTCCCGCTTTCATAAGAAAATATT-3'	pDG1729-PcshA
pDG1729-cshA-H	5'-ATCAAGCTTATAAAACTGCCCTTTAGA--3'	pDG1729-PcshA
695	5'-GCTTGTAAATTCTATCATAATTG-3'	Inverse PCR
696	5'-AGGGAATCATTTGAAGGTTGG-3'	Inverse PCR
ypuN-F	5'AAGAAGCTTCCGGCAGTTAAAGACC--3'	pMUTIN3DZ-ypuN
ypuN-R	5'-GGAGGATCCTTTTCAGGAACGACAAAC-3'	pMUTIN3DZ-ypuN
pX-cshA-seq-F	5'-CTTGTTCACTTAAATCAAAGG-3'	Sequence confirmation
pX-cshA-seq-R	5'-TTTCTGAATAATTTTGTCCCC-3'	Sequence confirmation
