# Supplementary material for: Glucose Induces ECF Sigma Factor Genes, sigX and sigM, Independent of Cognate Anti-sigma Factors through Acetylation of CshA in Bacillus subtilis
Source: Front Microbiol. 2016 Nov 29;7:1918. doi: 10.3389/fmicb.2016.01918 (PMC5126115; doi:10.3389/fmicb.2016.01918)
Supplement: Supplementary file 2 [file Image_1.PDF]

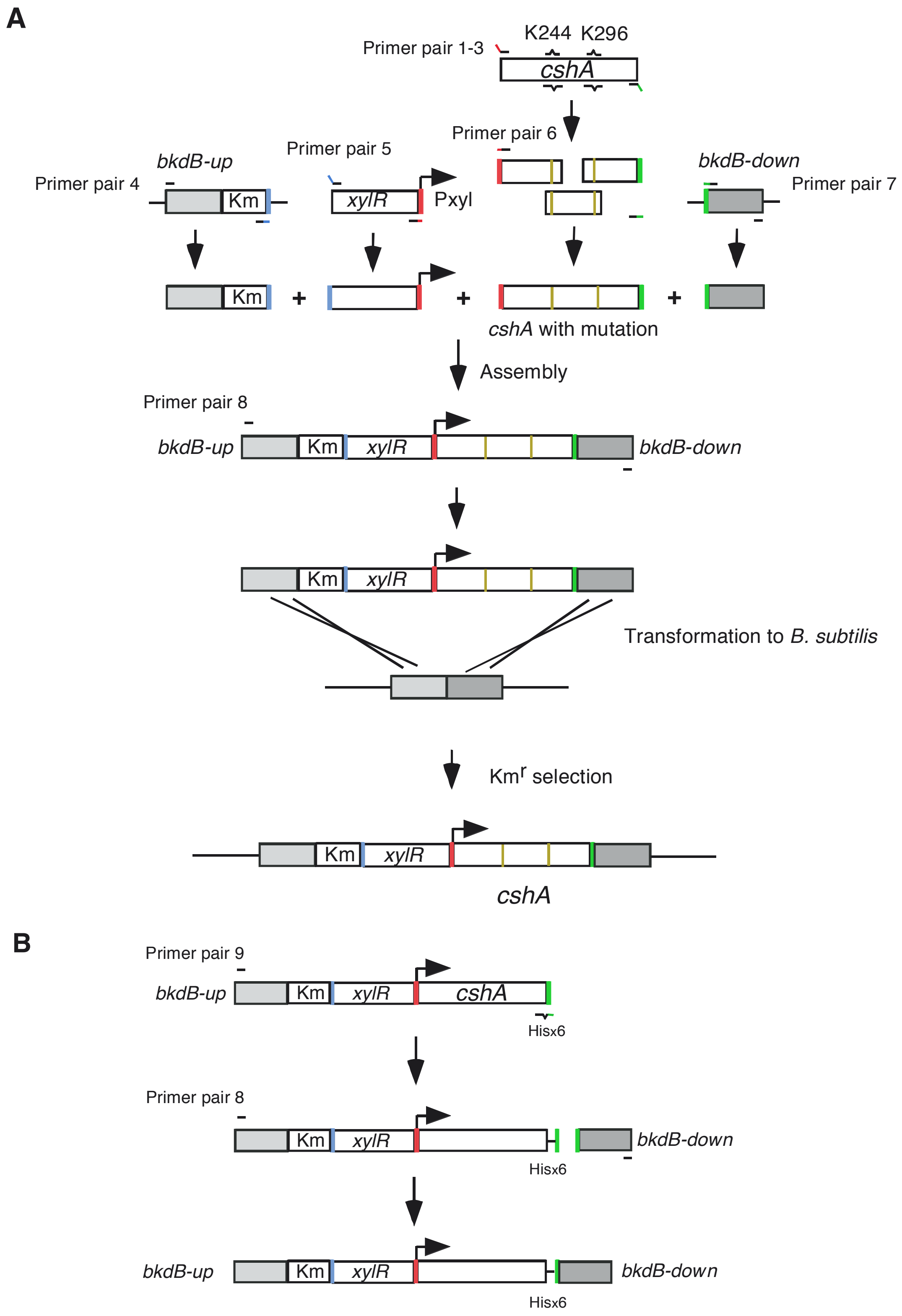

Figure S1. Schematic representation of the construction of *bkdB*::Pxyl-*cshA*. Arrows that are not associated with text indicate that PCR was performed. Primer pairs for each PCR are shown in Table S2. Boxes and bent arrows show open-reading frames and promoters, respectively. The same colored regions indicate that they have the same nucleotide sequences. (A) Construction scheme for wild type and mutant *cshA*. For the wild type gene, PCR with the primer pairs 1, 2, and 3 was not performed, instead the primer pair 6 was adopted. (B) Construction scheme for *cshA* encoding a His-tagged protein.
