# Supplementary material for: Glucose Induces ECF Sigma Factor Genes, sigX and sigM, Independent of Cognate Anti-sigma Factors through Acetylation of CshA in Bacillus subtilis
Source: Front Microbiol. 2016 Nov 29;7:1918. doi: 10.3389/fmicb.2016.01918 (PMC5126115; doi:10.3389/fmicb.2016.01918)
Supplement: Supplementary file 3 [file Image_2.PDF]

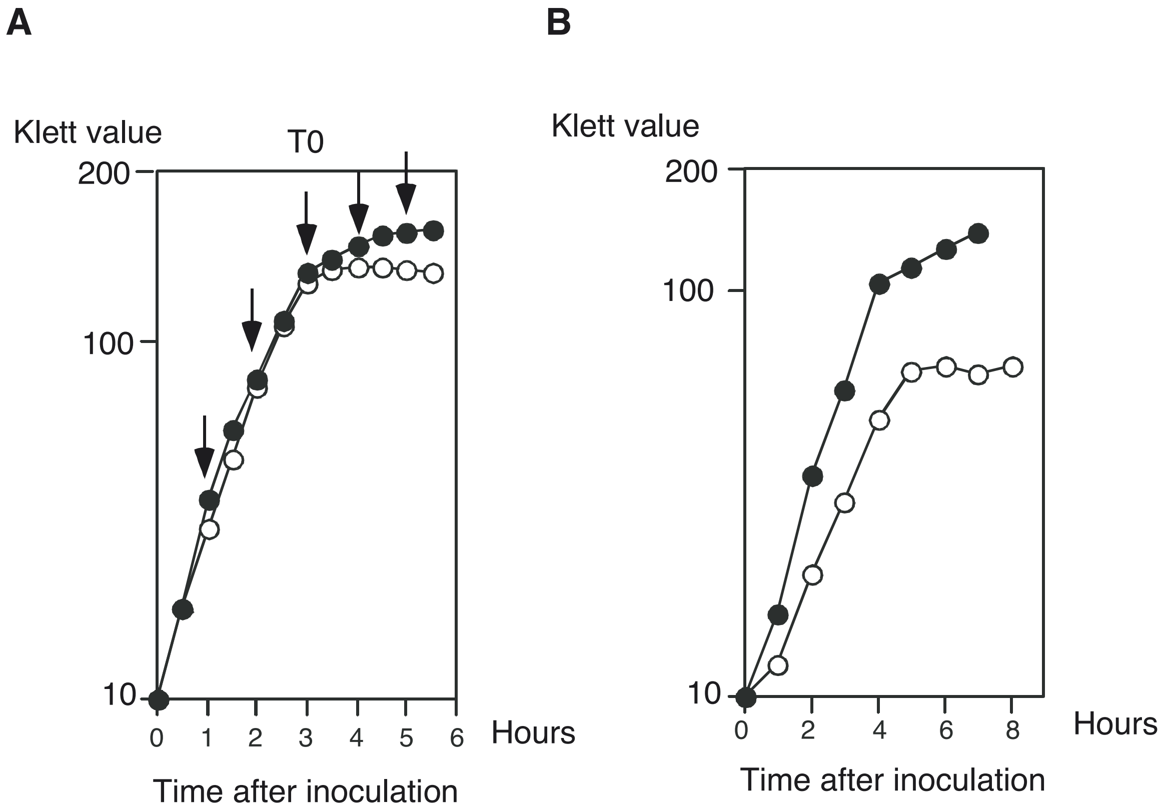

Figure S2. Growth of the strains. Growth curves of OAM692 (PyjbC-*lacZ,* A) and OAM716 (*sigM-lacZ pdhC*, B) monitored by Klett colorimeter are shown. Cells were grown in sporulation medium with (closed symbols) or without (open symbols) 2% glucose. The arrows above the curve in (A) show the sampling points for *lacZ* analysis.
