# Supplementary material for: Glucose Induces ECF Sigma Factor Genes, sigX and sigM, Independent of Cognate Anti-sigma Factors through Acetylation of CshA in Bacillus subtilis
Source: Front Microbiol. 2016 Nov 29;7:1918. doi: 10.3389/fmicb.2016.01918 (PMC5126115; doi:10.3389/fmicb.2016.01918)
Supplement: Supplementary file 4 [file Image_3.PDF]

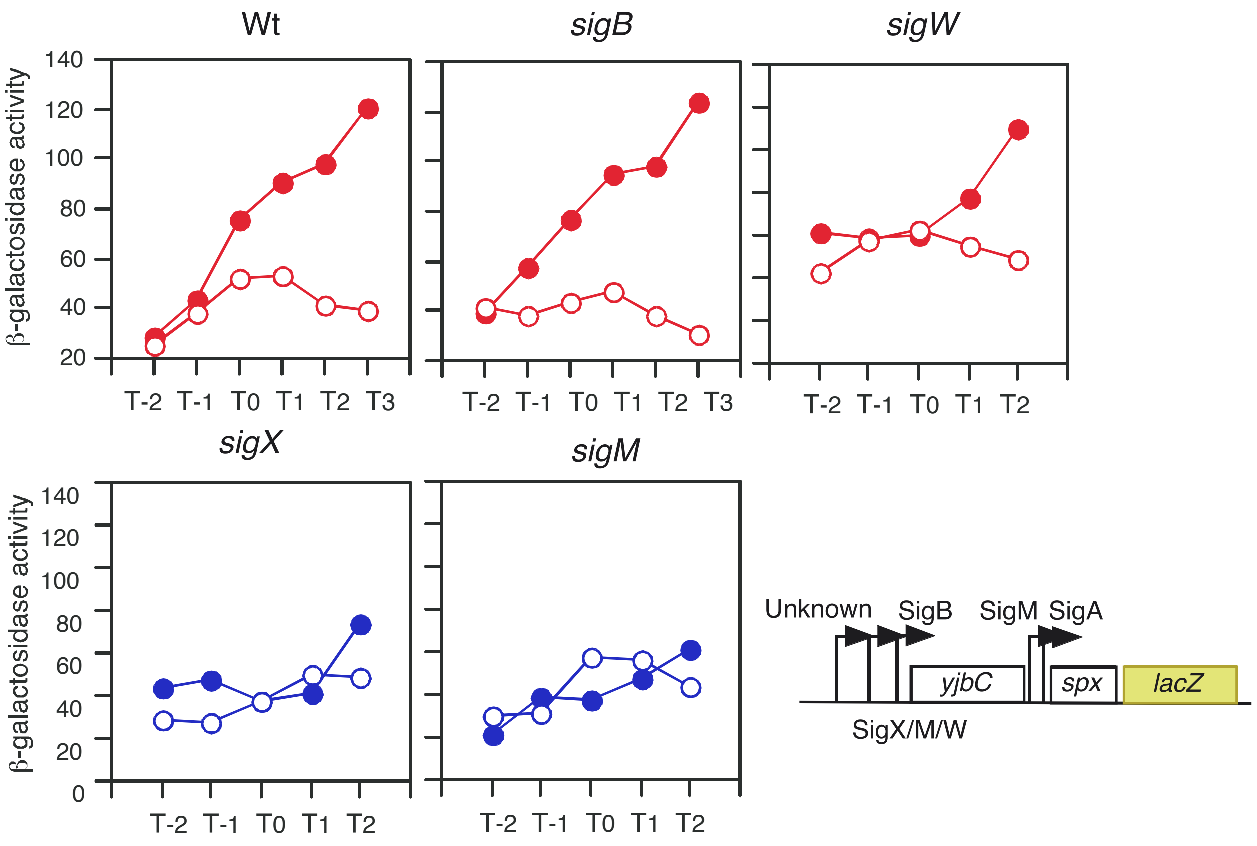

Figure S3. Abolishment of GI of *spx-lacZ* in *sigX* and *sigM* mutants. Cells were grown in sporulation medium with (closed symbols) or without (open symbols) 2% glucose. Cells were sampled hourly. β-galactosidase activities are shown in Miller units. The X-axis is the same as that in Fig 1. Data sets showing GI and not showing GI are shown in red and blue, respectively. The strain with the wild type fusion is BSF2842 and the introduced gene disruption is indicated above the panel. The chromosomal structure of *spx-lacZ* is shown. Boxes and bent arrows show open-reading frames and promoters, respectively. Text along with the bent arrow show the σ factors responsible for the promoter activity. Typical results are shown.
