# Supplementary material for: Glucose Induces ECF Sigma Factor Genes, sigX and sigM, Independent of Cognate Anti-sigma Factors through Acetylation of CshA in Bacillus subtilis
Source: Front Microbiol. 2016 Nov 29;7:1918. doi: 10.3389/fmicb.2016.01918 (PMC5126115; doi:10.3389/fmicb.2016.01918)
Supplement: Supplementary file 5 [file Image_4.PDF]

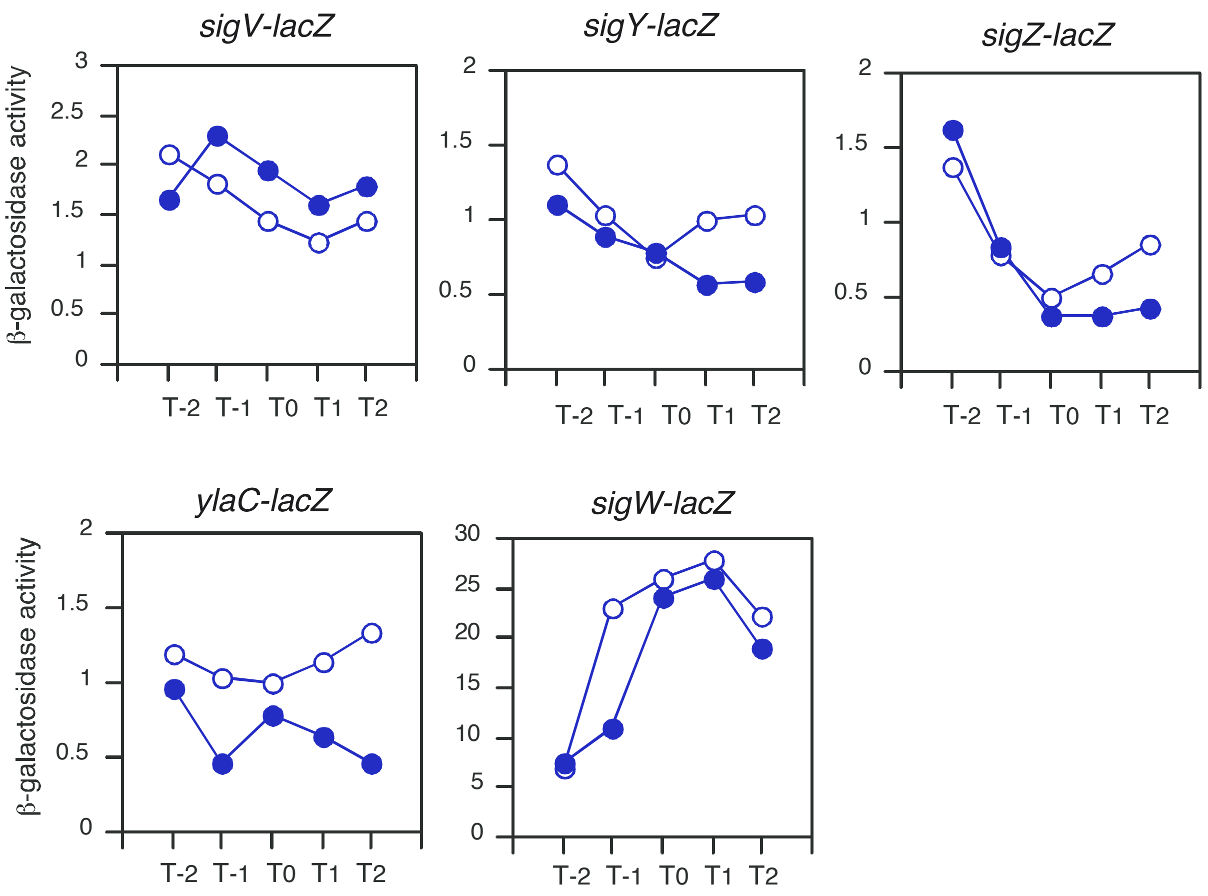

Figure S4. No GI of genes encoding other ECF σ factors. Cells were grown in sporulation medium with (closed symbols) or without (open symbols) 2% glucose. Cells were sampled hourly. β-galactosidase activities are shown in Miller units. The X-axis is the same as that in Fig 1. Each gene fusion is indicated above the panel. Typical results are shown.
