# Supplementary material for: Glucose Induces ECF Sigma Factor Genes, sigX and sigM, Independent of Cognate Anti-sigma Factors through Acetylation of CshA in Bacillus subtilis
Source: Front Microbiol. 2016 Nov 29;7:1918. doi: 10.3389/fmicb.2016.01918 (PMC5126115; doi:10.3389/fmicb.2016.01918)
Supplement: Supplementary file 6 [file Image_5.PDF]

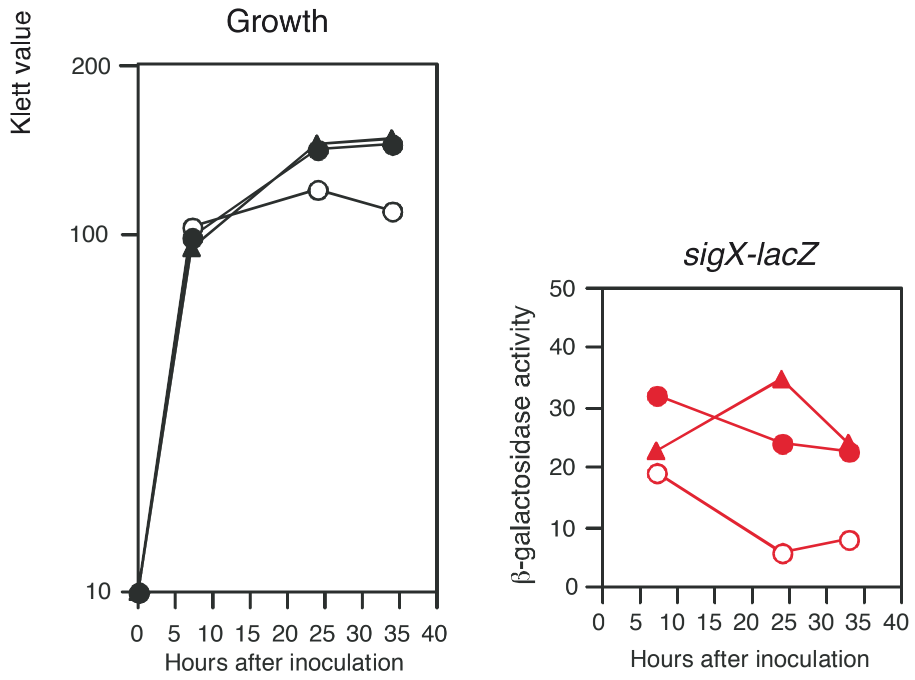

Figure S5. GI of *sigX-lacZ* in LB medium. Left and right panels show growth and β-galactosidase activities, respectively. OAM709 were grown in LB medium without glucose (open circles), with 1% (closed circles) and 2% glucose (closed triangles). Cells were sampled three times. β-galactosidase activities from *thrC*::*sigX-lacZ* driven only by σ^X-RNAP are shown in Miller units. The X-axis represents the growth time. A data set showing GI is shown in red. Typical results are shown. In our experimental condition, *aprE* expression begins at 7 h after inoculation to LB medium (Ogura and Tsukahara, 2010). This initiation indicates the entry into the early stationary phase.
