# Supplementary material for: Glucose Induces ECF Sigma Factor Genes, sigX and sigM, Independent of Cognate Anti-sigma Factors through Acetylation of CshA in Bacillus subtilis
Source: Front Microbiol. 2016 Nov 29;7:1918. doi: 10.3389/fmicb.2016.01918 (PMC5126115; doi:10.3389/fmicb.2016.01918)
Supplement: Supplementary file 8 [file Image_7.PDF]

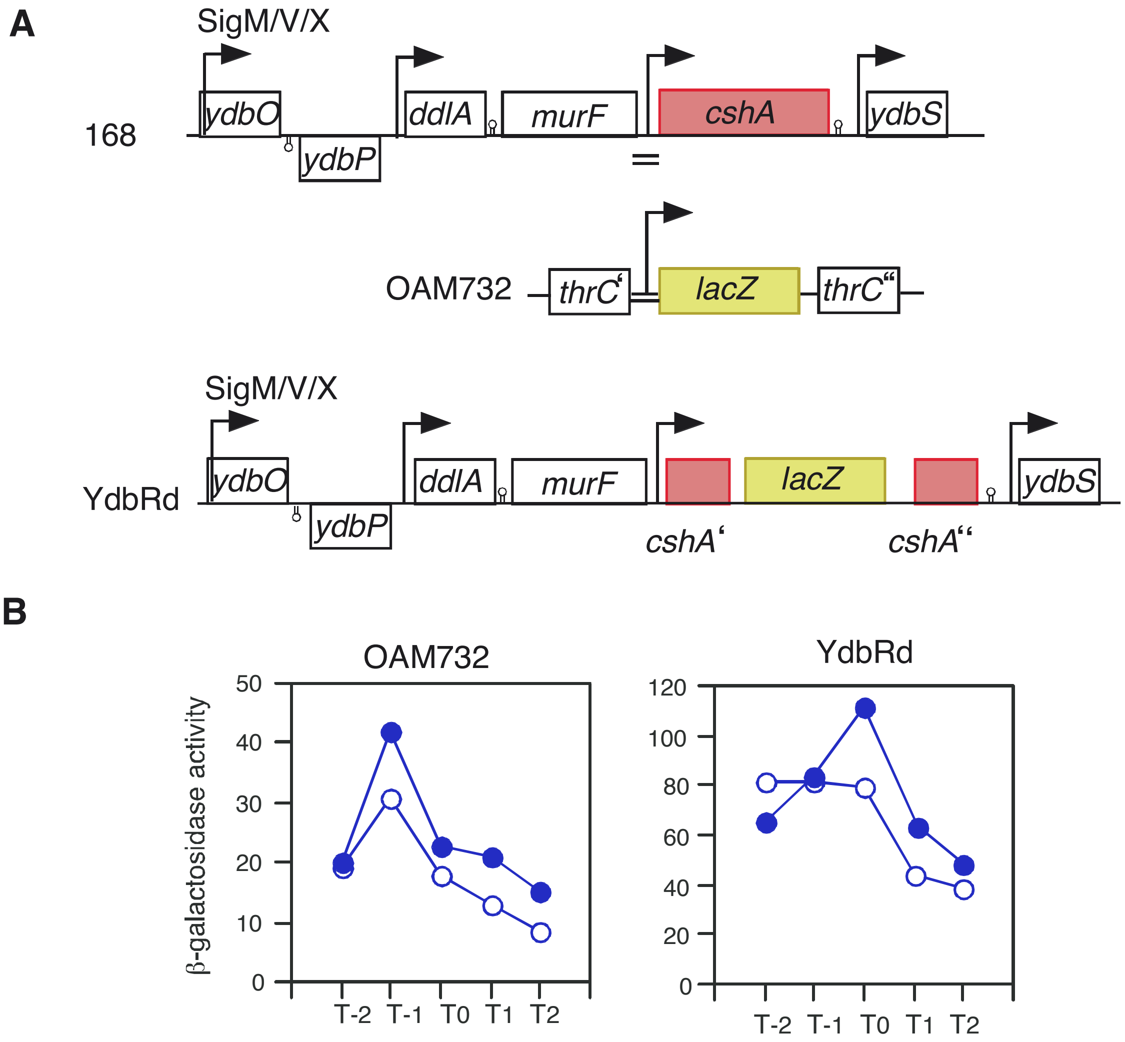

Figure S7. Expression of *cshA*. (A) The chromosomal structures of the *cshA* region and two strains, OAM732 and YdbRd. Boxes and bent arrows show open-reading frames and promoters, respectively. The stem-loop structure represents a terminator. Text along with the bent arrow show the σ factors responsible for the promoter activity. Double line indicates the cloned *cshA* promoter region into pDG1729-PcshA. (B) Cells were grown in sporulation medium without (open symbols) or with (closed symbols) 2% glucose. Cells were sampled hourly. β-galactosidase activities are shown in Miller units. The X-axis is the same as that in Fig 1. Data sets not showing GI are shown in blue. Left panel, circles, OAM732 (Wt). Right panel, YdbRd. Typical results are shown.
